# Update: COVID-19 Pandemic–Associated Changes in Emergency Department Visits — United States, December 2020–January 2021

**DOI:** 10.15585/mmwr.mm7015a3

**Published:** 2021-04-16

**Authors:** Jennifer Adjemian, Kathleen P. Hartnett, Aaron Kite-Powell, Jourdan DeVies, Roseric Azondekon, Lakshmi Radhakrishnan, Katharina L. van Santen, Loren Rodgers

**Affiliations:** ^1^CDC COVID-19 Response Team; ^2^ICF International Inc., Atlanta, Georgia.

During March 29–April 25, 2020, emergency department (ED) visits in the United States declined by 42% after the declaration of a national emergency for COVID-19 on March 13, 2020. Among children aged ≤10 years, ED visits declined by 72% compared with prepandemic levels (*1*). To assess the continued impact of the COVID-19 pandemic on EDs, CDC examined trends in visits since December 30, 2018, and compared the numbers and types of ED visits by patient demographic and geographic factors during a COVID-19 pandemic period (December 20, 2020–January 16, 2021) with a prepandemic period 1 year earlier (December 15, 2019–January 11, 2020). After an initial decline during March–April 2020 (*1*), ED visits increased through July 2020, but at levels below those during the previous year, until December 2020–January 2021 when visits again fell to 25% of prepandemic levels. During this time, among patients aged 0–4, 5–11, 12–17, and ≥18 years, ED visits were lower by 66%, 63%, 38%, and 17%, respectively, compared with ED visits for each age group during the same period before the pandemic. Differences were also observed by region and reasons for ED visits during December 2020–January 2021; more visits during this period were for infectious diseases or mental and behavioral health–related concerns and fewer visits were for gastrointestinal and upper-respiratory–related illnesses compared with ED visits during December 2019–January 2020. Although the numbers of ED visits associated with socioeconomic factors and mental or behavioral health conditions are low, the increased visits by both adults and children for these concerns suggest that health care providers should maintain heightened vigilance in screening for factors that might warrant further treatment, guidance, or intervention during the COVID-19 pandemic.

Data were obtained from the National Syndromic Surveillance Program (NSSP),* a collaborative system developed and maintained by CDC, state and local health departments, and academic and private sector health partners. NSSP collects electronic health data in near real-time, including ED visits from a subset of hospitals in 49 states (all but Hawaii) and the District of Columbia. This study analyzed information collected from approximately 71% of nonfederal facilities, nationwide, using data for all ED visits from participating hospitals in the 46 states that reported ED visits consistently during the prepandemic (December 15, 2019–January 11, 2020) and pandemic (December 20, 2020–January 16, 2021) periods assessed. All hospitals in Hawaii, Ohio, South Dakota, and Wyoming, and hospitals in other states that started or stopped reporting during 2019–2021 were excluded. Patient diagnoses were analyzed using a subset of records that included at least one specific, billable *International Classification of Diseases, Tenth Revision, Clinical Modification* (ICD-10-CM) code. Facilities that did not report diagnostic codes consistently or reported incomplete codes during 2019–2021 were excluded. ED visits were categorized using the Clinical Classifications Software Refined tool from the Healthcare Cost and Utilization Project, which combines ICD-10-CM codes into clinically meaningful groups (*2*).

This analysis was limited to the top 200 diagnostic categories (pediatric = 455 total diagnostic categories; adult = 497 total diagnostic categories) for each patient-level category evaluated during the assessed periods. The 10 categories with the highest and lowest significant (p<0.05) prevalence ratios (PRs)^†^ were identified. Trends in ED visits during December 30, 2018–January 16, 2021 were examined; overall analysis of trends focused on the prepandemic period during December 15, 2019–January 11, 2020 and the pandemic period during December 20, 2020–January 16, 2021, with comparisons by patient sex, age, U.S. Department of Health and Human Services (HHS) region,^§^ and reason for the visit. Estimates of weekly change^¶^ and PRs were calculated to assess differences in numbers of ED visits between the two periods. All analyses were conducted using R software (version 4.0.; The R Foundation) This activity was reviewed by CDC and was conducted consistent with applicable federal law and CDC policy.**

After decreasing by 42% during March–April 2020 (*1*), overall U.S. ED visits increased through July 2020 then stabilized in August 2020 at levels 15% below those during the same prepandemic period. During December 2020–January 2021, numbers of visits declined again to a level 25% lower than those during the previous year (December 2019–January 2020) (Figure), including a 23% decline in visits by males and a 27% decline in visits by females. During December 2020–January 2021, the numbers of ED visits in all age groups were lower than were those during the prepandemic period. The largest observed decline in visits was among children, especially those aged 0–4 years (66%) and 5–11 years (63%) (Supplementary Figure 1, https://stacks.cdc.gov/view/cdc/104808). ED visits by adults aged ≥18 years were 17% lower than ED visits during the prepandemic period (Figure). During December 2020–January 2021, ED visits varied by HHS region, ranging from an overall 29% decrease in the upper Midwest to a 21% decrease in the Northeast. ED visits by adults and pediatric patients declined in all regions (Supplementary Figure 2, https://stacks.cdc.gov/view/cdc/104808), ranging from a 23% decrease in the West (Region 9) to a 14% decrease in the Northeast (Region 3) among adults, and from 65% in the Northeast (Region 2) to 53% in the Midwest (Region 7) among children.

**FIGURE:**
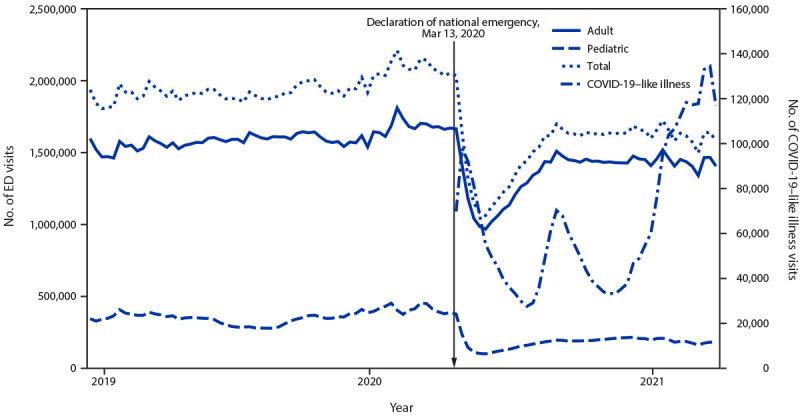
Weekly number of total,* adult,^†^ and pediatric^§^ emergency department (ED) visits and COVID-19–like illness visits — National Syndromic Surveillance Program, United States,^¶^ December 30, 2018–January 16, 2021 * Total, adult, and pediatric visits include visits for COVID-19–like illness. ^†^ Patients aged ≥18 years. ^§^ Patients aged <18 years. ^¶^ Forty-six states and the District of Columbia. All facilities in Hawaii, Ohio, South Dakota, and Wyoming, and facilities in other states that started or stopped reporting to the National Syndromic Surveillance Program during 2019–2021 were excluded.

During December 2020–January 2021, the proportion of ED visits for infectious disease–related concerns (i.e., exposure, encounters, screening, or contact with infectious disease) was higher than that during the same period before the pandemic for adults (PR = 5.86) and children (PR = 9.22), as were the proportion of visits related to socioeconomic and psychosocial (mental and behavioral health–related concerns) factors (adults PR = 1.37; children PR = 2.56). Among adults, the proportion of ED visits during this period was also higher than that during the prepandemic period for menopausal disorders (PR = 1.89); respiratory failure, insufficiency, and arrest (PR = 1.62); acute pulmonary embolism (PR = 1.59); cardiac arrest and ventricular fibrillation (PR = 1.45); malaise and fatigue (PR = 1.34); acute and unspecified renal failure (PR = 1.33); and symptoms of mental and substance-use conditions (PR = 1.28) (Table 1). Among children, the proportion of ED visits during this period was higher compared with the prepandemic period for calculus of the urinary tract (PR = 2.70); open wounds to limbs, subsequent encounter (PR = 2.67); suicidal ideation, attempt, and intentional self-harm (PR = 2.64); sexually transmitted infections (HIV and viral hepatitis) (PR = 2.57); schizophrenia spectrum and other psychotic disorders (PR = 2.55); lifestyle and life management factors (e.g., tobacco use, lack of physical exercise, high-risk sexual behavior, sleep deprivation or insomnia, or stress or burnout) (PR = 2.55); feeding and eating disorders (PR = 2.52); and open wounds of the head and neck, subsequent encounter (PR = 2.51) (Table 2). Decreases in the proportion of ED visits related to gastrointestinal and upper respiratory–related factors were identified in both adults and children, with the largest declines among children for influenza (PR = 0.01), acute bronchitis (PR = 0.17), pneumonia except that caused by tuberculosis (PR = 0.30), otitis media (0.36), and sinusitis (PR = 0.42).

**TABLE 1:** Prepandemic to pandemic* changes in the number of weekly emergency department (ED) visits^†^ among adults aged ≥18 years and prevalence ratios (PRs),^§^ by diagnostic categories^¶^ with the highest and lowest PRs** — National Syndromic Surveillance Program (NSSP), United States,^††^ December 15, 2019–January 16, 2021

Diagnostic category	Absolute change in mean no. of weekly ED visits	PR (95% CI)
**Highest PRs**		
Exposure, encounters, screening, or contact with infectious disease	54,570	5.86 (5.81–5.92)
Menopausal disorders	1,789	1.89 (1.85–1.93)
Respiratory failure, insufficiency, and arrest	6,884	1.62 (1.61–1.64)
Acute pulmonary embolism	1,056	1.59 (1.55–1.62)
Cardiac arrest and ventricular fibrillation	601	1.45 (1.42–1.49)
Socioeconomic/Psychosocial factors	878	1.37 (1.35–1.39)
Malaise and fatigue	2,605	1.34 (1.33–1.35)
Acute and unspecified renal failure	2,317	1.33 (1.32–1.34)
Symptoms of mental and substance use conditions	239	1.28 (1.25–1.30)
Abnormal findings without diagnosis	2,227	1.27 (1.26–1.28)
**Lowest PRs**		
Influenza	−34,870	0.03 (0.03–0.03)
Acute bronchitis	−21,984	0.26 (0.26–0.27)
Sinusitis	−8,227	0.41 (0.40–0.42)
Otitis media	−4,945	0.41 (0.41–0.42)
Other specified upper respiratory infections	−33,488	0.48 (0.48–0.49)
Intestinal infection	−2,398	0.62 (0.61–0.64)
Cornea and external disease	−3,258	0.70 (0.69–0.71)
Noninfectious gastroenteritis	−5,944	0.71 (0.70–0.72)
Viral infection	−9,986	0.74 (0.73–0.75)
Other specified and unspecified disorders of the ear	−3,394	0.75 (0.74–0.76)

**Abbreviation**: CI = confidence interval.

* Prepandemic period analyzed was December 15, 2019–January 11, 2020; pandemic period analyzed was December 20, 2020–January 16, 2021.

^†^ The weekly change in ED visits during the pandemic and comparison prepandemic periods was calculated as the difference in total counts between the two periods, divided by 4 weeks. Absolute change in mean number of ED visits for each diagnostic category is presented as a data label within parentheses. In the pandemic period, the average weekly visits across facilities for adults was 403 (range = 0.25–5,906) and in the comparison period, the average weekly visits across facilities for adults was 493 (range = 0.25–11,756).

^§^ Ratio calculated as the proportion of all ED visits in each diagnostic category during the pandemic period, divided by the proportion of all ED visits in that category during the comparison period. Ratios >1 indicate a higher proportion of visits in that category during the pandemic period than during the comparison period; ratios <1 indicate a lower proportion during the pandemic period than during the comparison period.

^¶^ ED visits were categorized using the Clinical Classifications Software Refined tool from the Healthcare Cost and Utilization Project, which combines *International Classification of Diseases, Tenth Revision, Clinical Modification* codes into clinically meaningful groups. https://www.hcup-us.ahrq.gov/toolssoftware/ccsr/dxccsr.jsp

** The analysis was limited to the top 200 diagnostic categories for each patient-level category (pediatric = 455 total diagnostic categories; adult = 497 total diagnostic categories) evaluated during the assessed period. The 10 categories with the highest and lowest significant (p<0.05) PRs were identified.

^††^ Only facilities consistently reporting informative discharge diagnoses in the two periods (i.e., not null and without terms like “unknown”) were included in the analysis. Forty-six states and the District of Columbia are included. All facilities in Hawaii, Ohio, South Dakota, and Wyoming, and facilities in other states that started or stopped reporting to the NSSP during 2019–2021 were excluded.

**TABLE 2:** Prepandemic to pandemic* changes in the number of weekly emergency department (ED) visits^†^ among children aged <18 years and prevalence ratios (PRs),^§^ by diagnostic categories^¶^ with the highest and lowest PRs** — National Syndromic Surveillance Program (NSSP), United States,^††^ December 15, 2019–January 16, 2021

Diagnostic category	Absolute change in mean no. of weekly ED visits	PR (95% CI)
**Highest PRs**		
Exposure, encounters, screening, or contact with infectious disease	6,175	9.22 (9.01–9.43)
Calculus of urinary tract	18	2.70 (2.44–2.98)
Open wounds to limbs, subsequent encounter	9	2.67 (2.34–3.06)
Suicidal ideation/attempt/intentional self-harm	174	2.64 (2.57–2.72)
Sexually transmitted infections (excluding HIV and hepatitis)	5	2.57 (2.26–2.94)
Socioeconomic/Psychosocial factors	22	2.56 (2.41–2.72)
Lifestyle/Life management factors	12	2.55 (2.36–2.76)
Schizophrenia spectrum and other psychotic disorders	6	2.55 (2.27–2.86)
Feeding and eating disorders	2	2.52 (2.18–2.92)
Open wounds of head and neck, subsequent encounter	4	2.51 (2.26–2.79)
**Lowest PRs**		
Influenza	−33,554	0.01 (0.01–0.01)
Acute bronchitis	−15,308	0.17 (0.16–0.17)
Pneumonia (except that caused by tuberculosis)	−5,665	0.30 (0.29–0.31)
Otitis media	−20,187	0.36 (0.35–0.36)
Sinusitis	−1,085	0.42 (0.39–0.45)
Other specified upper respiratory infections	−43,194	0.48 (0.48–0.48)
Cornea and external disease	−3,900	0.51 (0.49–0.52)
Viral infection	−21,378	0.53 (0.52–0.53)
Intestinal infection	−1,726	0.58 (0.56–0.61)
Diseases of middle ear and mastoid (except otitis media)	−486	0.62 (0.57–0.67)

**Abbreviation**: CI = confidence interval.

* Prepandemic period analyzed was December 15, 2019–January 11, 2020; pandemic period analyzed was December 20, 2020–January 16, 2021.

^†^ The weekly change in ED visits during the pandemic and comparison prepandemic periods was calculated as the difference in total counts between the two periods, divided by 4 weeks. Absolute change in mean number of ED visits for each category in the figures is presented as a data label within parentheses. In the pandemic period, the average weekly visits across facilities for pediatrics was 50 (range = 0.25–1,316) and in the comparison period the average weekly visits across facilities for pediatrics was 122 (range = 0.25–2,565).

^§^ Ratio calculated as the proportion of all ED visits in each diagnostic category during the pandemic period, divided by the proportion of all ED visits in that category during the comparison period. Ratios >1 indicate a higher proportion of visits in that category during the pandemic period than during the comparison period; ratios <1 indicate a lower proportion during the pandemic period than during the comparison period.

^¶^ ED visits were categorized using the Clinical Classifications Software Refined tool from the Healthcare Cost and Utilization Project, which combines *International Classification of Diseases, Tenth Revision, Clinical Modification* codes into clinically meaningful groups. https://www.hcup-us.ahrq.gov/toolssoftware/ccsr/dxccsr.jsp

** The analysis was limited to the top 200 diagnostic categories for each patient-level category (pediatric = 455 total diagnostic categories; adult = 497 total diagnostic categories) evaluated during the assessed period. The 10 categories with the highest and lowest significant (p<0.05) PRs were identified.

^††^ Only facilities consistently reporting informative discharge diagnoses in the two periods (i.e., not null and without terms like “unknown”) were included in the analysis. Forty-six states and the District of Columbia are included. All facilities in Hawaii, Ohio, South Dakota, and Wyoming, and facilities in other states that started or stopped reporting to the NSSP during 2019–2021 were excluded.

## Discussion

After a decline in ED visits in the United States associated with the COVID-19 pandemic during March–April 2020 (*1*), ED visits steadily increased through July 2020, and then stabilized through the fall. During December 2020–January 2021, visits declined again to a level 25% lower than that during December 2019–January 2020. These declines were highest in children aged ≤10 years, who had 65% fewer ED visits during December 2020–January 2021 than during December 2019–January 2020. Although ED visits increased among adults during December 2020–January 2021, they were 17% below those during the prepandemic period. There was a decline in ED visits among children for conditions such as influenza, acute bronchitis, and pneumonia, which could reflect reduced transmission of other pathogens; therefore the decreased visits might represent appropriate use of ED care or that children might be disproportionately affected by changes in care-seeking behaviors because of the COVID-19 pandemic. The reasons for ED visits have changed during the pandemic period compared with those during the prepandemic period. More visits were associated with severe respiratory and cardiovascular conditions during the pandemic period; more adults and children have also been seeking emergency care for mental or behavioral health and socioeconomic and psychosocial concerns. However, weekly numbers for visits for some categories of mental or behavioral health diagnoses (e.g., feeding and eating disorders) remain relatively low, particularly among pediatric patients.

Decreases in the numbers of ED visits among children might disproportionately affect families that lack reliable access to primary care and might instead use EDs for treatment (*3*), possibly preventing them from obtaining needed care. In addition, the wide regional variations in numbers of ED visits might indicate differences in public health messaging and risk perceptions regarding COVID-19, stay-at-home policies, transmission patterns, access to testing and primary care, as well as other factors. Possible barriers to necessary medical care should be addressed with targeted public health messaging and clinical guidance to ensure that treatment for critical conditions is not delayed. Although the numbers of ED visits associated with socioeconomic factors and mental or behavioral health conditions are low, the increased proportion of these visits by both adults and children suggests that health care providers should maintain heightened vigilance in screening for factors that might warrant further treatment, guidance, or intervention during the COVID-19 pandemic (*4*,*5*).

The findings in this report are subject to at least four limitations. First, diagnostic categories rely on the use of specific codes, which might be missing or used inconsistently across hospitals (*6*). Second, NSSP coverage is not uniform across or within states; some hospitals report statewide and others do not report statewide or have no data available for some counties. However, given that NSSP data represent 71% of U.S. EDs, trends identified at the national level likely represent actual patterns in persons seeking care during the COVID-19 pandemic. Third, this analysis did not analyze NSSP data by age, sex, race, and ethnicity within each region; future studies that evaluate this information can help guide interventions to address the increased prevalence of socioeconomic factors and mental or behavioral health conditions associated with ED visits. Finally, ED visits and trends are likely the result of many factors that can be challenging to fully understand with limited patient data available; additional studies are needed to help guide public health communication strategies on ED use.

These findings provide updates for clinical and public health stakeholders on the changing profile of ED visits associated with the COVID-19 pandemic. CDC is available to provide support to sites interested in participating in NSSP to monitor for critical trends in ED visits. As the nation continues to manage the impact of the ongoing pandemic, public understanding of the importance of seeking guidance and emergency care for acute and mental or behavioral health conditions is necessary. Wider access to health messages, triage help lines, and virtual visits that help all persons, especially caregivers of children and adolescents, can help determine when seeking immediate care might be warranted and might also result in fewer patients seeking ED care (*7*).

**Summary**

**What is already known about this topic?**

During March 29–April 25, 2020, U.S. emergency department (ED) visits declined by 42% after the declaration of a national emergency for COVID-19 on March 13, 2020. The number of ED visits increased by July 2020 but remain below prepandemic levels.

**What is added by this report?**

ED visits during December 2020–January 2021 were 25% lower than during the same months the year before. Higher proportions of ED patients are seeking care for mental and behavioral health–related concerns, especially pediatric patients.

**What are the implications for public health practice?**

Efforts to ensure public understanding of the importance of seeking guidance and emergency care for acute and mental or behavioral health conditions are necessary.

## Footnotes

*
https://www.cdc.gov/nssp/index.html

† PR and associated 95% confidence interval of visits was calculated for each diagnostic category as the proportion of ED visits during the pandemic period (December 20, 2020–January 16, 2021) divided by the proportion of visits during the comparison prepandemic period (December 15, 2019–January 11, 2020) ([ED visits in diagnostic category in pandemic period/all ED visits in pandemic period]/[ED visits in diagnostic category in comparison period/all ED visits in comparison period]).

§ HHS Region 1: Connecticut, Maine, Massachusetts, New Hampshire, Rhode Island, and Vermont; Region 2: New Jersey, New York. Region 3: Delaware, District of Columbia, Maryland, Pennsylvania, Virginia, and West Virginia; Region 4: Alabama, Florida, Georgia, Kentucky, Mississippi, North Carolina, South Carolina, and Tennessee; Region 5: Illinois, Indiana, Michigan, Minnesota, and Wisconsin; Region 6: Arkansas, Louisiana, New Mexico, Oklahoma, and Texas; Region 7: Iowa, Kansas, Missouri, and Nebraska; Region 8: Colorado, Montana, North Dakota, and Utah; Region 9: Arizona, California, Nevada; Region 10: Alaska, Idaho, Oregon, and Washington.

¶ The weekly change in ED visits during the pandemic and comparison prepandemic periods was calculated as the difference in total counts between the two periods, divided by 4 weeks ([visits (pandemic period) in diagnostic category − visits (comparison period) in diagnostic category]/4).

** 45 C.F.R. part 46, 21 C.F.R. part 56; 42 U.S.C. Sect. 241(d); 5 U.S.C. Sect. 552a; 44 U.S.C. Sect. 3501 et seq.
